# Do Black Cats Look Less Adoptable? Human Judgments of Emotions and Adoptability in Online Shelter Photographs in the United States

**DOI:** 10.3390/ani16060869

**Published:** 2026-03-11

**Authors:** Jill A. Villarreal, Reese Gebauer, James C. Ha

**Affiliations:** 1 Department of Animal Behavior, Stephens College, Columbia, MO 65215, USA; 2 Montessori Adolescence Program, Saint Louis, MO 63108, USA; 3 Department of Psychology, University of Washington, Seattle, WA 98195, USA

**Keywords:** cat, color, adoption, perception, emotion

## Abstract

Each year, millions of cats enter U.S. animal shelters, where black cats experience lower adoption rates. This study examined whether three factors contribute to this disparity: (1) superstitious beliefs, (2) projected skin-color bias, and (3) difficulty interpreting black cats’ emotions in online photographs sourced from *Petfinder*, an online searchable, adoption database. A total of 1004 U.S.-based participants completed a 10 min online survey in which they viewed 40 cat adoption advertisement photos: 10 each of black, white, orange tabby, and brown tabby cats. For each image, participants selected one of six basic emotions, rated their confidence, and judged the likelihood that the cat would be adopted within two weeks. Additional items measured superstition and skin-color bias. Black cats were rated as significantly less adoptable than other cats. Participants also attributed negative emotions, such as fear and anger, to black cats more frequently and reported lower confidence when interpreting their emotional expressions. Superstition and skin-color bias, however, did not significantly affect adoptability rating or emotional perception. These findings suggest that difficulty interpreting black cats’ emotional cues in photographs may increase perceptual uncertainty, which in turn influences judgments of adoptability.

## Simple Summary

Many animal shelters report that black cats are adopted less often and spend more time waiting for homes than cats of other colors. This study explored three possible reasons for this pattern: whether people hold superstitious beliefs about black cats, whether people project attitudes about human skin color onto cats based on their coat color, and whether people have difficulty reading black cats’ emotions in online photographs. Over one thousand adults viewed adoption images of cats from *Petfinder*, an online, searchable adoption database. Participants rated the cats’ emotions, their confidence in those judgments, and how likely each cat was to be adopted within the next two weeks. Black cats were rated as less likely to be adopted, were more often described as looking fearful or angry, and were harder for people to “read” with confidence. These results suggest that difficulty interpreting facial expressions and body language in photographs of black cats may contribute to lower adoption interest. Improving lighting, contrast, and image clarity, and helping potential adopters focus on each cat’s individual personality, may help black cats find homes more quickly.

## 1. Introduction

Each year in the United States, approximately 3.2 million cats are admitted to animal shelters, according to data from the American Society for the Prevention of Cruelty to Animals [1]. Of these, an estimated 530,000 are euthanized, while approximately 2.1 million are adopted into homes. Research consistently indicates that black cats experience longer stays in shelters, lower adoption rates, and higher euthanasia risk compared to cats of other coat colors [2,3,4,5,6]. Studies examining adoption outcomes have identified physical characteristics, particularly coat color, as influential factors [4,7], suggesting that public perception plays a meaningful role in these disparities.

Jones and Hart [8] found that individuals rated black cats as less friendly, more aggressive, and less adoptable than cats of other colors, despite no evidence of behavioral differences associated with black coat coloration. Such stereotypes may significantly influence adoption decisions, especially given that friendliness is one of the most highly valued traits among potential adopters [9,10]. Contributing factors may include cultural superstitions [8,11] as well as broader societal attitudes related to skin-color bias that could extend from human social perception to animals.

Across many cultures, the color black carries negative symbolic associations, including death, grief, evil, and the unknown [12,13,14,15]. Folklore frequently portrays black cats as omens of bad luck or as symbols of sin and wickedness [12,13,16], and historical narratives have linked black cats to witchcraft, including beliefs that witches could transform into black cats [17]. Despite these cultural narratives, there is no scientific evidence indicating that black cats differ behaviorally from cats of other coat colors.

Beyond superstition, broader misconceptions about cats may influence how potential adopters interpret feline emotions. Cats are often anthropomorphized, with their behaviors and facial expressions interpreted through a human emotional framework that may not accurately reflect feline affective states. Bouma, Reijgwart, and Dijkstra [18] found that cat owners who described their relationship with their cat in human terms (e.g., child or best friend) were more likely to attribute complex social emotions, such as jealousy or compassion, and to assign emotions like sadness or curiosity to neutral cat photographs. Similarly, Vonk and Bouma [19] demonstrated that interpretations of cats’ emotions are influenced by the strength of owner attachment, with stronger attachment associated with greater attribution of secondary emotions. Croney et al. [20] further emphasized that cat welfare is closely tied to human perceptions of cat emotions.

These misunderstandings are reinforced by cultural narratives that portray cats, particularly certain coat colors, as emotionally distant, unpredictable, or less socially affiliative than other companion animals. Such beliefs may shape adopters’ expectations and evaluations, especially when judgments are based on static images rather than direct interaction. When potential adopters view online shelter photographs, these perceptual biases may be amplified. In the absence of behavioral context, individuals may rely on preconceived beliefs to infer emotional states and adoptability. This may disproportionately disadvantage cats whose facial features or expressions are more difficult to visually discern, such as black cats.

Another possible source for the different views on black cats may be the anthropomorphic projection of skin tone beliefs about people based on skin onto cats based on their coat color. Research regarding human biases has explored the concept of colorism, which refers to the preferential treatment of human individuals with lighter skin tones and the corresponding marginalization of those with darker complexions [21]. The “bad is black” assumption has also been studied, showing that people were more likely to place blame for immoral acts on those with darker skin [22]. Colorism operates under the assumption that lighter (often white) skin color in humans is linked to social dominance, privilege, and higher socioeconomic status [23]. This form of bias has historically targeted communities of color and continues to manifest in both subtle and overt forms of discrimination today [24,25]. For instance, African Americans with darker skin have been shown to earn lower incomes, on average, than their lighter-skinned counterparts [26]. Delgado, Munera, and Reevy [27] reported that white cats were more frequently perceived as “aloof.” Aloofness in humans is often culturally associated with authority and elevated social status [28,29,30]. Consistent with this, research on social perception indicates that high-status groups are often perceived as highly competent but low in warmth, a pattern commonly described as the “competent but cold” stereotype. Low warmth includes traits such as emotional distance or aloofness, whereas high competence is associated with dominance and authority. Perceptions of aloofness in lighter-colored cats may reflect the anthropomorphic transfer of human status-based stereotypes, wherein emotional distance signals competence and authority, while darker coloration may be implicitly associated with more negative emotions.

Workman and Hoffman [31] reported that approximately one-third of adopters they surveyed had visited Petfinder prior to adopting, and half of those had viewed the Petfinder profile of the cat they ultimately adopted. Petfinder is an online adoption platform connecting potential adopters with nearly 11,000 animal shelters and rescue organizations across the United States [32]. With the continued rise in digital technologies, it is likely that an increasing number of adopters rely on photo-based online searches when seeking a cat. However, shelter staff and volunteers frequently report difficulty capturing high-quality photographs of black cats that are visually engaging and emotionally clear to potential adopters.

Although cats have been documented to produce nearly 300 distinct facial signals [33], it has been suggested that dark, monochromatic coat coloration may make emotional cues more difficult to perceive in photographs. Facial expressions are critically important for human emotion recognition, with certain expressions considered universal across cultures [34]. When a cat’s facial features are unclear or poorly lit, potential adopters seeking a friendly or approachable animal may hesitate. Workman and Hoffman [31] noted this challenge when examining the impact of coat color on shelter cat adoption rates, suggesting that less visually appealing online profiles may contribute to lower adoption outcomes for black cats. Schoenfeld-Tacher, Kogan, and Carney [35] similarly proposed that photographic images, particularly of black cats, may inadequately represent personality traits, thereby influencing adoption decisions. Caeiro, Burrows, and Waller [36] further suggested that darker-coated cats’ facial features are more difficult to discern, which may affect judgments of emotion and adoptability.

In the present study, we examined three proposed factors that may contribute to lower adoption rates and longer shelter stays for black cats: superstitious beliefs, skin-color bias attitudes, and difficulty interpreting emotional cues from photographs. First, we hypothesized that individuals reporting higher levels of superstition would rate black cats as less adoptable. Second, we hypothesized that individuals reporting higher levels of skin-color bias would rate black cats as less adoptable and attribute more negative emotions to them. Finally, we hypothesized that participants would report lower confidence when judging the emotions of black cats compared to cats of other coat colors.

## 2. Method

### 2.1. Participants

Ethical approval for this study was obtained from the Institutional Review Board of Harris-Stowe State University, and all procedures were conducted in accordance with approved protocols. The study employed a convenience sampling approach, and participants self-selected into the survey. Participants responded to an online survey distributed through *SurveyMonkey* by email, social media, print fliers, and word of mouth between 3 May 2024, and 7 March 2025. Eligible participants were required to be at least 18 years old and reside in the United States. Of the 1004 participants who completed the survey, 313 (31.3%) were between the ages of 18–24, 173 (17.3%) were 25–34, 173 (17.3%) were 35–44, 169 (16.9%) were 45–54, 95 (9.5%) were 55–64, 59 (5.9%) were 65–74, 18 (1.8%) were 75–84, 2 (0.0%) were 85 years or above, and 2 (0.0%) did not wish to disclose their age. In terms of gender identity, seven-hundred and fifteen participants identified as female (71.2%), 255 as male (25.4%), 20 as another gender (2.0%), and 14 chose not to disclose their gender (1.4%). Regarding racial and ethnic identity, 483 (48.1%) participants identified as Black or African American, 342 (34.1%) as White or Caucasian, 70 (7.0%) as Asian or Asian American, 58 (5.8%) as Hispanic or Latino/a, 16 (1.6%) as another ethnicity, 10 (1.0%) as American Indian or Native American, 5 (0.5%) as Native Hawaiian or Pacific Islander, and 20 (2.0%) chose not to disclose their ethnicity.

Household income levels varied among participants, with 93 (9.3%) reporting an annual income of less than $10,000, 58 (5.8%) reporting between $10,000 and $20,000, 123 (12.2%) reporting between $21,000 and $40,000, 138 (13.7%) reporting between $41,000 and $60,000, 121 (12.0%) reporting between $61,000 and $80,000, 86 (8.6%) reporting between $81,000 and $100,000, and 258 (25.7%) reporting an income greater than $100,000. Additionally, 127 (12.6%) participants chose not to disclose their income. Participants were from 39 states, with 55% residing in Missouri, 16% in Illinois, 5% in California, 3% in Florida, 3% in Texas, and 2% in New York. Ohio accounted for 1% of participants, while all other states had ten or fewer participants. Among participants, 420 (41.8%) identified their cat knowledge as novice, 139 (13.8%) as advanced beginner, 213 (21.2%) as competent, 156 (15.5%) as proficient, and 76 (7.6%) as expert.

When asked if they had lived with a cat, 632 (62.9%) participants reported having lived with a cat, while 372 (37.1%) had not. Regarding their attitude toward cats, 332 (33.1%) participants strongly agreed with the statement “I love cats”, 197 (19.6%) agreed, 244 (24.3%) neither agreed nor disagreed, 113 (11.3%) disagreed, and 118 (11.8%) strongly disagreed.

### 2.2. Survey

The survey’s first section served as the participants’ informed consent. Information about the research team conducting the survey and the purpose of the study was provided. This section stated that the psychology research lab invited participants to take part in a voluntary, survey-based study that would take approximately 10 min to complete. It also stated that there was no financial compensation for participation. Potential participants were informed that all responses were anonymous and that participants’ names and contact information were never recorded. It was also indicated that, although a secure system was used to collect the data, the risk that individuals’ online data could be hacked or intercepted when using the internet could not be eliminated. Potential participants were informed that a substantial number of participants were needed to observe patterns accurately and that the reported data would be analyzed and reported in aggregate rather than as individual responses. This section also stated that the psychology lab would collect, analyze, and report the data. The data would then be stored at the university for up to two years. The Institutional Review Board of Harris-Stowe State University could review all study data to ensure that applicable laws and ethical guidelines were followed. The final part of this section stated that participants were required to be 18 years of age or older and to currently reside in the United States. Potential participants were provided with the principal investigator’s email address for questions. The final line of this section was where participants provided their consent to participate in the survey.

The second section of the survey included demographic information questions. They were asked their age, zip code, gender identification, ethnic or racial identification, annual household income, cat behavior knowledge, if they have ever lived with a cat, and if they have or have had a career involving cats. They also rated how strongly they agreed with the statement “I love cats” on a 5-point Likert scale ranging from strongly agree (score of 5) to strongly disagree (score of 1).

**Figure 1:**
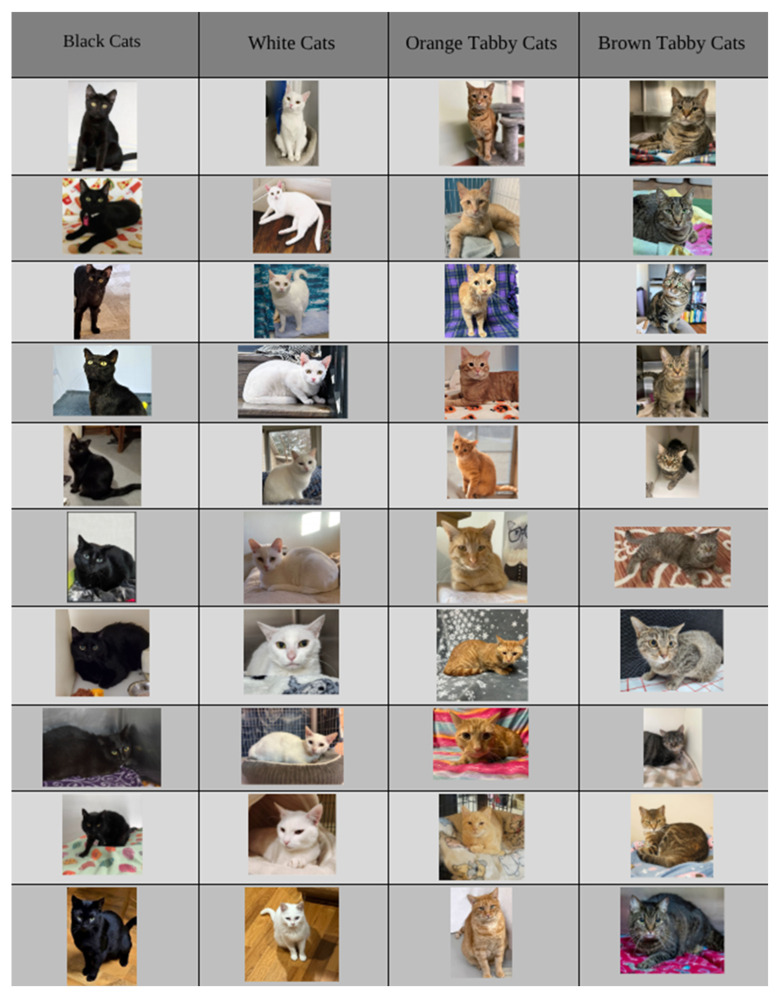
Chart of photos rated by participants. Photos were presented to participants in random order.

The third section of the survey showed participants a series of 40 cat photographs retrieved from www.Petfinder.com between January to February 2024 from cat adoption postings across shelters (See Figure 1). Images were selected from cat adoption posting across shelters throughout the United States to reflect photographs typically viewed by potential adopters during online searches. Inclusion criteria required that each image clearly displayed a single cat with a visible face, relatively neutral background, and sufficient lighting to allow facial features to be discerned. Images containing humans, toys, costumes, filters, text overlays, or multiple animals were excluded. Across coat color categories, efforts were made to control for additional visual factors that could influence emotion perception, including body position, head orientation, ear position, and visible whisker orientation. While complete standardization was not possible given the use of real shelter photographs, images were chosen to approximate comparable visual presentations across coat colors to the extent feasible. The final image set consisted of 40 photographs, including 10 black, 10 white, 10 orange tabby, and 10 brown tabby cats, presented in random order (see Figure 1).

After each cat image, participants were asked to judge which basic emotion they felt best described the cat in the picture. Participants were instructed that if they were unsure, to give their best guess. The basic emotion choices provided to select from were: Angry, Disgusted, Fearful, Sad, Happy, and Surprised. These choices were from Ekman and Friesen [34] and represented emotions studied in people, not cats. We chose to use human emotional labels, as opposed to feline traits described in the feline literature, because we felt the humanistic language would be more relatable to the general US population. After they selected an emotion, they were asked to rate how confident they were in their judgement using a 5-point scale: 1 = Not confident at all, 2 = Not so confident, 3 = Somewhat confident, 4 = Very Confident, and 5 = Extremely confident. Next, they were asked if they thought the cat would be adopted in the next two weeks using a Likert scale: 1 = Definitely no, 2 = Probably no, 3 = Not sure, 4 = Probably yes, 5 = Definitely yes. The two-week adoption window was selected to reflect the high-throughput practices common in many U.S. animal shelters, where adoption or euthanasia decisions often occur within a short timeframe due to pet overpopulation. In many animal control shelters, cats may have only a few weeks to secure adoption. The next section of the survey covered superstitious beliefs and colorist opinions. First, participants were asked to report whether they had engaged in behaviors motivated by superstition beliefs. Statements included were selected from previously published indexes of superstition [37,38]. Participants rated the six statements below on a 5-point scale: 1 = Definitely no, 2 = Probably no, 3 = Not sure, 4 = Probably yes, and 5 = Definitely yes.

1. Avoided walking under a ladder because it is associated with bad luck?
2. Felt anxious about breaking a mirror because it is thought to cause bad luck?
3. Felt anxious about a black cat crossing your path because it is thought to cause bad luck?
4. Felt superstitious about any number, such as 13 or 4?
5. Said ‘touch wood’ or actually touched or knocked on wood to avoid bad luck?
6. Carried a lucky charm or object?

Next, participants rated five statements about skin-tone attitudes, using a 5-point Likert scale: 1 = Strongly disagree, 2 = Disagree, 3 = Neither agree nor disagree, 4 = Agree, 5 = Strongly agree.

The statements were selected from research by Harvey, Tennial, and Hudson Banks [39]. We did not include their entire skin-tone attitude index to keep the needed time to complete the survey to around 10 min. The statements included were as follows:

1. Skin tone plays a big part in determining how far you can make it.
2. Dark-skinned people are more difficult to work with.
3. I usually choose who I’m going to be friends with by their skin tone.
4. My skin tone is a big part of my identity.
5. Lighter skin tone makes others more attractive.

Then participants were asked if they identified as religious (Protestantism, Catholicism, Christianity, Judaism, Islam, Buddhism, Hinduism, Native American, Inter/Non-denominational, Wiccan, or Other) or non-religious. They were then asked who referred them to the survey, to ensure that our student research assistants were equally contributing to the collection of the survey sample. Next was a comment box for participants to write in any comments. The survey concluded by providing the email address of the principal investigator for any additional comments or concerns. Participants were also thanked for completing the survey.

### 2.3. Procedure

This study was approved by the Harris-Stowe State University Institutional Review Board. The survey was generated and tested with the help of two high school students, research interns from the Montessori Adolescent Program-St. Louis. The survey was distributed from 3 May 2024 to 7 March 2025. The survey was distributed online using a convenience sampling method.

### 2.4. Analysis

Participants who did not complete the entire survey were withdrawn from the dataset. For the remaining participants, we calculated a superstition index score by taking the mean for their rating of the six statements about superstition. We also calculated a skin-tone attitudes index score by taking the mean for their rating of the five statements about skin-tone attitudes. The resulting independent factors described above were used to test hypotheses focusing on the differential perception of emotion, confidence in emotion rating, and adoptability of cats of different coat colors using a Multivariable General Linear Model to help account for inherent statistical relations within the predictor variables. Individual group differences in all tests were established by visual inspection of confidence intervals and Tukey’s post hoc pairwise comparisons. The alpha for all tests was set at 0.05. In the case of multiple comparisons, a Bonferroni correction was applied. All tests were conducted in SYSTAT v13.

## 3. Results

### 3.1. Adoptability Ratings

Perceived adoptability differed significantly by coat color, with black cats having significantly lower adoptability scores, (*F*(3, 3009) = 566.92, *p* = 0.001: Black *M* = 2.931 (*SE* = 0.018), Orange *M* = 3.453 (*SE* = 0.018), White *M* = 3.349 (*SE* = 0.018), Brown *M* = 3.387 (*SE* = 0.018).

Participant age impacted adoptability ratings, only when evaluating black cats, (*F*(21, 2982) = 1.795, *p* = 0.02). Younger participants reported on average they were “not sure” if the black cats shown would be adopted in the next two weeks; whereas, older age groups’ mean ratings fell within the “probably not” range.

Gender differences were found only for orange tabby cats, (*F*(6, 2958) = 3.656, *p* = 0.002). Participants who self-identified as male gave orange tabby cats a mean adoptability rating of 3.363 (*SE* = 0.37); whereas participants who self-identified as female gave orange tabby cats a slightly higher adoptability rating of 3.484 (*SE* = 0.021).

Income groups differed in their rating of the adoptability of black cats, (*F*(21, 2988) = 2.655, *p* = 0.001), with participants reporting annual incomes over $100,000 rating black cats as having slightly lower adoptability.

Participants’ superstition index score showed no relation with the selected perceived emotional state of cats with different coat colors. There was also no relation found between a skin-tone attitudes index score and perception of emotional state in cats of differing coat colors.

### 3.2. Perceived Emotional State

#### 3.2.1. Angry

Perceived emotional state varied across coat colors. Black cats were perceived more often as angry and orange tabby cats perceived less often as angry than brown tabby cats or white cats. Six independent *t*-tests were conducted with *df* = 1003 and t-values ranging from −0.894 (*NS*) to 17.709. Mean anger rating were as follows: Black cats *M* =1.371 (*SE* = 0.034), Orange tabby cats *M* = 0.075 (*SE* = 0.33), White cats *M* = 1.022 (*SE* = 0.037), Brown tabby cats *M* = 1.054 (*SE* = 0.034).

#### 3.2.2. Fearful

Black cats were rated as appearing fearful more often than all other coat colors, followed by brown tabby cats, orange tabby cats, and white cats. All pairwise comparisons were significant. Six independent *t*-tests were conducted with *df* = 1003 and t-values ranging from 3.331 to 12.022. Mean fear ratings were as follows: Black cats *M* = 2.269 (*SE* = 0.047), Orange tabby cats *M* = 1.953 (*SE* = 0.043), White cats *M* = 1.665 (*SE* = 0.046) and Brown tabby cats *M* = 2.110 (*SE* = 0.045).

#### 3.2.3. Sad

Orange tabby and white cats were rated as appearing sad more often than cats of other coat colors, followed by black cats, whereas brown tabby cats were rated as sad the least often. Six independent *t*-tests were conducted with *df* = 1003 and t-values ranging from 1.898 (*NS*) to 16.856. Mean sadness ratings were as follows: Black cats *M* =1.039 (*SE* = 0.037), Orange tabby cats *M* = 1.663 (*SE* = 0.043), White cats *M* = 1.574 (*SE* = 0.044), Brown tabby cats *M* = 0.904 (*SE* = 0.035).

### 3.3. Confidence in Emotional Ratings

Participants reported lower confidence in their ratings of emotional states for black cats than other coat colors, (*F*(3, 3009) = 296.116, *p* = 0.001: Black cats *M* = 2.955 (*SE* = 0.022), Orange tabby cats *M* = 3.196 (*SE* = 0.023), White cats *M* = 3.165 (*SE* = 0.023), Brown tabby *M* = 3.221 (*SE* = 0.024).

There was a significant relationship between the degree to which participants self-reported as “loving cats” and their confidence in rating the images for their emotional state, (*F*(12, 2997) = 3.896, *p* = 0.001). Participants who “strongly agreed” that they love cats reported greater confidence across coat colors. Those who selected “agree,” “neither agree nor disagree,” “disagree,” or “strongly disagree” did not significantly differ from one another.

## 4. Conclusions

Results indicate that black cats were perceived as less adoptable than cats of other coat colors. When shown photographs of black cats, participants were less likely to agree that the cat would be adopted within the next two weeks compared to white, orange tabby, or brown tabby cats. Participants in the present study attributed negative emotions, particularly fear and anger, to black cats more frequently than to cats of other coat colors. Participants also reported lower confidence when interpreting the emotional expressions of black cats. Contrary to our expectations, two of the three central hypotheses were not supported. We did not find evidence that lower perceived adoptability of black cats was directly associated with self-reported superstition or with skin-tone bias attitudes.

## 5. Discussion

The present findings point to systematic differences in how observers interpret the emotional expressions of cats across coat colors. Participants more frequently attributed negative human emotions, particularly fear and anger, to black cats and reported lower confidence in interpreting their emotional states. These perceptual uncertainties and negative attributions provide a plausible psychological context for the continued adoption disadvantage documented for black cats in shelter statistics [2,3,4,5,6,7,8,9,10]. When considered alongside prior work showing longer lengths of stay and lower adoption rates for black cats, the current results suggest that ambiguity and negativity in emotion perception may be one mechanism through which visual appearance shapes expectations about adoptability.

Contrary to our initial hypotheses, individual differences in self-reported superstitious beliefs and skin-tone bias attitudes were not associated with perceived adoptability judgments. This absence of a relationship warrants careful consideration. One possibility is that superstition and skin-color bias operate implicitly rather than through consciously endorsed beliefs, making them less detectable using self-report measures. Prior research suggests that superstitious thinking is widespread and often maintained even when individuals recognize such beliefs as unfounded, because these beliefs may arise from fast, automatic (Type I) cognitive processes [40,41,42,43]. Similarly, research indicates that individuals are often not consciously aware of their own skin-tone biases, and that tools such as the Implicit Association Tests may be better suited to detecting such attitudes. Individuals may also distance themselves from overtly superstitious or biased beliefs while still responding to culturally embedded associations at a perceptual level.

Generational shifts and increased public awareness may further reduce explicit endorsement of superstition or skin-tone bias, even if residual effects persist in behavior or judgment. Ertit [44] noted a decline over time in the social prestige and influence of folk belief, magic, and the supernatural. Likewise, Kurdi, Charlesworth, and Mair [45] analyzed data from 33 countries between 2009 and 2019 and reported substantial reductions in self-reported (explicit) bias across multiple domains, including race, skin tone, and body weight, despite continued variability in implicit bias. Contemporary social norms may discourage open endorsement of biased beliefs, thereby limiting the sensitivity of self-report measures. Future research should therefore consider incorporating implicit measures to more fully capture the influence of superstition and color-based bias.

The phrasing of the adoptability question represents an additional limitation. Participants were asked to judge whether a cat would be adopted within the next two weeks, rather than whether they themselves would be interested in adopting the cat. As a result, participants may have based their responses on expectations about other adopters’ behavior rather than their own preferences. This distinction may partially explain the absence of associations between adoptability ratings and individual belief measures. Future studies should directly compare responses to questions assessing personal adoption intent versus perceived likelihood of adoption by others.

Consistent with prior research [8], participants in the present study attributed negative emotions, particularly fear and anger, to black cats more frequently than to cats of other coat colors. Participants also reported lower confidence when interpreting the emotional expressions of black cats. These findings may reflect the difficulty of capturing clear emotional cues in photographs of dark, monochromatic animals. Adopters tend to seek cats that appear happy and socially responsive [8,42], and when emotional signals are ambiguous, potential adopters may experience greater uncertainty, which may lower perceived adoptability. Although photographic features were not experimentally manipulated in this study, the observed associations among emotion attribution, confidence, and perceived adoptability provide a basis for generating testable hypotheses and guiding future applied research.

Several limitations related to generalizability should be acknowledged. All participants resided in the United States, and over half were located in Missouri, with additional clustering in a small number of Midwestern and coastal states. Consequently, the observed perceptions of black cat adoptability reflect attitudes embedded within a specific sociocultural context shaped by U.S. shelter practices, media representations, and prevailing cultural narratives. Adoption norms, superstitious beliefs, and interpretations of animal emotion may differ across cultural and geographic settings, particularly in regions where black cats are viewed more positively or where shelter systems operate under different ethical or structural frameworks. These findings should therefore not be generalized beyond the regions sampled without caution.

Generalizability is further constrained using convenience sampling and the absence of stratification by key sociodemographic variables, such as race, income, education, or prior shelter adoption experience. Cultural, social, and regional norms surrounding companion animals and adoption practices may meaningfully influence how cats are perceived and evaluated. Accordingly, the present findings should be interpreted as reflecting patterns within a specific convenience sample rather than as representative of the broader U.S. population. Future research employing stratified, representative, or cross-cultural designs would strengthen the external validity of these findings.

In addition to the factors examined here, other unmeasured visual and contextual variables may influence emotion perception and adoptability judgments. Subtle differences in coat patterning, eye visibility, body posture, head orientation, and tail position may affect the ease with which emotional cues are interpreted from photographs. Photographic context, including background contrast, lighting angle, camera distance, and whether a cat is depicted inside a kennel or in a more naturalistic environment, may further shape perception. Although efforts were made to approximate comparable visual presentations across images, these factors were not systematically manipulated or quantified. Future research should isolate and experimentally test the relative contribution of these visual and contextual features to more precisely identify the mechanisms underlying coat-color-related disparities.

Despite these limitations, the present findings suggest several promising directions for future applied research and shelter-based trials aimed at supporting black cats. If potential adopters experience greater uncertainty when interpreting black cats’ emotions in photographs, interventions designed to enhance visual clarity may warrant evaluation. Such approaches could include improved lighting, high-contrast backgrounds, inviting bedding or toys, and brief photography guides or toolkits for shelter staff and volunteers. Training volunteers to recognize and capture “adoption-friendly” moments, such as relaxed postures or playful interactions, also represents a testable applied strategy. In addition, shelters may consider evaluating the use of video alongside still photographs. Research on adoptable dogs suggests that video presentations are associated with more positive trait attributions [46]. Future studies should determine whether similar benefits extend to cats.

Beyond photographic presentation, shelters and rescue organizations may also play an important role in counteracting coat-color bias through intentional communication and policy practices. Emphasizing that no scientific evidence links a cat’s coat color to personality or behavior may help reduce reliance on appearance-based assumptions. Educational messaging integrated into adoption websites, social media, and in-shelter materials, along with clear behavior-based descriptions, foster notes, and brief temperament assessments, may shift attention away from visual ambiguity and toward individual characteristics. Adoption platforms may also benefit from evaluating whether standardizing profile content to prioritize behavioral information improves adoption-related decision making. Together, these strategies may support more informed adoption evaluations and help ensure that black cats are assessed based on their individual traits rather than stereotypes, thereby supporting improved adoption outcomes.

## Data Availability

The raw data from this study are not publicly available due to the conditions described in the informed consent process. Participants were informed that their responses would be analyzed and reported only as group patterns, stored for up to two years, and may be reviewed by the Institutional Review Board for ethical compliance. These conditions do not permit the sharing of individual-level data.

## References

[1] About Us/Press Release. https://www.aspca.org/about-us/press-releases/aspca-releases-new-data-showing-remarkable-progress-homeless-dogs-cats.

[2] Brown P.W., Morgan T.M. (2014). Age, breed, designation, coat color, and coat pattern influenced the length of stay of cats at a no-kill shelter. J. Appl. Anim. Welf. Sci..

[3] Dybdall K., Stasser R. (2014). Is there a bias against stray cats in shelters? People’s perception of shelter cats and how it influences adoption time. Anthrozoös.

[4] Lepper M., Kass P.H., Hart L.A. (2002). Prediction of adoption versus euthanasia among dogs and cats in a California animal shelter. J. Appl. Anim. Welf. Sci..

[5] Janke N., Berke O., Flockhart T., Bateman S., Coe J. (2017). Risk factors affecting length of stay of cats in an animal shelter: A case study at the Guelph Humane Society, 2011-2016. Prev. Vet. Med..

[6] Carini R.M., Sinski J., Weber J.D. (2020). Coat color and cat outcomes in an urban U.S. shelter. Animals.

[7] Kogan L.R., Schoenfeld-Tacher R., Hellyer P.W. (2013). Cats in animal shelters: Exploring the common perception that black cats take longer to adopt. Open Vet. J..

[8] Jones H.D., Hart C.L. (2020). Black cat bias: Prevalence and predictors. Psychol. Rep..

[9] Sinn L. (2016). Factors affecting the selection of cats by adopters. J. Vet. Behav..

[10] Southland A., Dowling-Guyer S., McCobb E. (2019). Effect of visitor perspective on adoption decisions at one animal shelter. J. Appl. Anim. Welf..

[11] Lockwood R. (2005). Cruelty toward cats: Changing perspectives. J. Am. Vet. Med. Assoc..

[12] Adams F.M., Osgood C.E. (1973). A cross-cultural study of the affective meanings of color. J. Cross-Cult. Psychol..

[13] Allan K. (2009). The connotations of English colour Terms: Colour-based X-phemisms. J. Pragmat..

[14] Kaya N., Epps H.H. (2004). Relationship between color and emotion: A study of college students. Coll. Stud. J..

[15] Sherman G.D., Clore G.L. (2009). The color of sin: White and black are perceptual symbols of moral purity and pollution. Psychol. Sci..

[16] If You Want to Adopt a Black Cat, You May Have to Wait Until Halloween Is Over. https://www.smithsonianmag.com/smart-news/if-you-want-adopt-black-cat-you-may-have-wait-until-halloween-is-over-180960868/.

[17] Linton R. (1951). Halloween. Sci. Am..

[18] Bouma E.M.C., van den Bos R., Vinke C.M. (2024). Cat owners’ anthropomorphic perceptions of feline emotions and interpretation of photographs. Appl. Anim. Behav. Sci..

[19] Vonk J., Bouma E.M.C. (2024). Attachment as the catalyst for the attribution of complex cognition and emotion to companion cats. Animals.

[20] Croney C.C., McCobb E., Widowski T.M. (2024). Debunking common myths about cats: Implications for feline welfare and human–cat interactions. Animals.

[21] Hunter M. (2007). The persistent problem of colorism: Skin tone, status, and inequality. Sociol. Compass.

[22] Alter A.L., Stern C., Granot Y., Balcetis E. (2016). The bad is black effect: Why people believe evildoers have darker skin than do-gooders. Pers. Soc. Psychol. Bull..

[23] Dixon A.R., Telles E.E. (2017). Skin color and colorism: Global research, concepts, and measurement. Annu. Rev. Sociol..

[24] Hunter M. (2013). The consequences of colorism. The Melanin Millennium.

[25] Monk E.P. (2015). The cost of color: Skin color, discrimination, and health among African Americans. Am. J. Sociol..

[26] Keith V.M., Herring C. (1991). Skin tone and stratification in the Black community. Am. J. Sociol..

[27] Delgado M., Munera D., Reevy M. (2012). Human perceptions of coat color as an indicator of domestic cat personality. Anthrozoös.

[28] Tsai J.L., Ang J.Y., Blevins E., Goernandt J., Fung H.H., Jiang D., Elliott A.J., Uchida Y. (2016). Leaders’ displays of emotional expressions signal dominance and prestige. J. Personal. Soc. Psychol..

[29] Fiske S.T., Dupree C.H., Nicolas G., Swencionis J.K. (2016). Status, power, and intergroup relations: The personal is the societal. Curr. Opin. Psychol..

[30] Fiske S.T., Cuddy A.J.C., Glick P., Xu J. (2002). A model of (often mixed) stereotype content: Competence and warmth respectively follow from perceived status and competition. J. Personal. Soc. Psychol..

[31] Workman M.K., Hoffman C.L. (2015). An evaluation of the role of coat color and sex on adoption rates of shelter cats. Anthrozoös.

[32] About Petfinder. https://www.petfinder.com/adopt-or-get-involved/about-petfinder/.

[33] Scott L., Florkiewicz B.N. (2023). Feline faces: Unraveling the social function of domestic cat facial signals. Behav. Process..

[34] Ekman P., Friesen W.V. (1971). Constants across cultures in the face and emotion. J. Pers. Soc. Psychol..

[35] Schoenfeld-Tacher R., Kogan L.R., Carney P.C. (2019). Perception of Cats: Assessing the differences between videos and still pictures on adoptability and associated characteristics. Front. Vet. Sci..

[36] Caeiro C.C., Burrows A.M., Walker B.M. (2017). Development and application of CatFACS: Are human cat adopters influenced by cat facial expressions?. Appl. Anim. Behav. Sci..

[37] Bridgstock M., Marais I., Sturgess K. (2011). The structure of superstitious action—A further analysis of fresh evidence. Pers. Individ. Dif..

[38] Fluke S., Webster R., Saucier D. (2014). Methodological and theoretical improvements in the study of superstitious beliefs and behaviour. Br. J. Psychol..

[39] Harvey R.D., Tennial R.E., Banks Hudson K. (2017). The development and validation of a colorism scale. J. Black Psychol..

[40] Risen J.L. (2015). Believing what we do not believe: Acquiescence to superstitious beliefs and other powerful intuitions. Psychol. Rev..

[41] Hall W.J., Chapman M.V., Lee K.M., Merino Y.M., Thomas T.W., Payne B.K., Eng E., Day S.H., Coyne-Beasley T. (2015). Implicit racial/ethnic and skin-tone bias among health care providers and associations with clinical care outcomes. Soc. Sci. Med..

[42] Crutchfield J., Sparks D., Williams M., Findley E. (2021). In my feelings: Exploring implicit skin tone bias among preservice teachers. J. Black Stud..

[43] Elvers G.C., Gavin B., Crutcher R.J. (2024). Explicit and implicit measures of black cat bias in cat and dog people. Animals.

[44] Ertit V. (2018). Secularization: The decline of the supernatural realm. Religions.

[45] Kurdi B., Charlesworth T.E.S., Mair P. (2025). International stability and change in explicit and implicit attitudes: An investigation spanning 33 countries, five social groups, and 11 years (2009–2019). J. Exp. Psychol. Gen..

[46] Pyzer C., Clarke L., Montrose V.T. (2017). Effects of video footage versus photographs on perception of dog behavioral traits. J. Appl. Anim. Welf. Sci..

